# Primary basal cell carcinoma of the prostate with concurrent adenocarcinoma

**DOI:** 10.1002/iju5.12143

**Published:** 2020-01-14

**Authors:** David Hennes, Adrian Dragovic, James Sewell, Meng Yeong Hoh, Richard Grills

**Affiliations:** ^1^ Department of Urological Surgery Barwon Health University Hospital Geelong Geelong Victoria Australia; ^2^ Department of Surgery Deakin University Geelong Victoria Australia

**Keywords:** adenocarcinoma, basal, carcinoma, cell, prostate

## Abstract

**Introduction:**

Prostatic basal cell carcinoma is an extremely rare tumor, exhibiting various histopathological features and clinical spectrums of disease.

**Case presentation:**

A 69‐year‐old male presented to our department with 2 years of voiding difficulty and intermittent macroscopic hematuria. With a presumed diagnosis of benign prostatic hyperplasia, he underwent a transurethral resection of the prostate. Pathological examination revealed atypical basaloid cells forming solid nests. Robot‐assisted radical prostatectomy was subsequently performed, confirming a diagnosis of basal cell carcinoma with coexisting acinar adenocarcinoma.

**Conclusion:**

Although more cases of basal cell carcinoma are indolent than aggressive, there is no reliable method of differentiation between these presentations. Thus, we recommend radical surgery and 6‐monthly disease surveillance until more is discovered about this very rare malignancy.

**Abbreviations & Acronyms**
MRI: magnetic resonance imaging
PBCC: prostatic basal cell carcinoma
PSA: prostate‐specific antigen
TURP: transurethral resection of the prostate

**Keynote message**

We herein report a case of PBCC, an exceptionally uncommon entity for which the optimal treatment remains unknown. The heterogonous clinical course of this tumor ranges from indolent to aggressive, with an inability to predict which patients are prone to metastasis or recurrence despite early radical treatment. Due to this variable malignant potential, and relative lack of effective treatment options, radical surgery and prudent longitudinal surveillance should be considered if basal cell carcinoma of the prostate is diagnosed at an early stage.

## Case presentation

**Figure 1:**
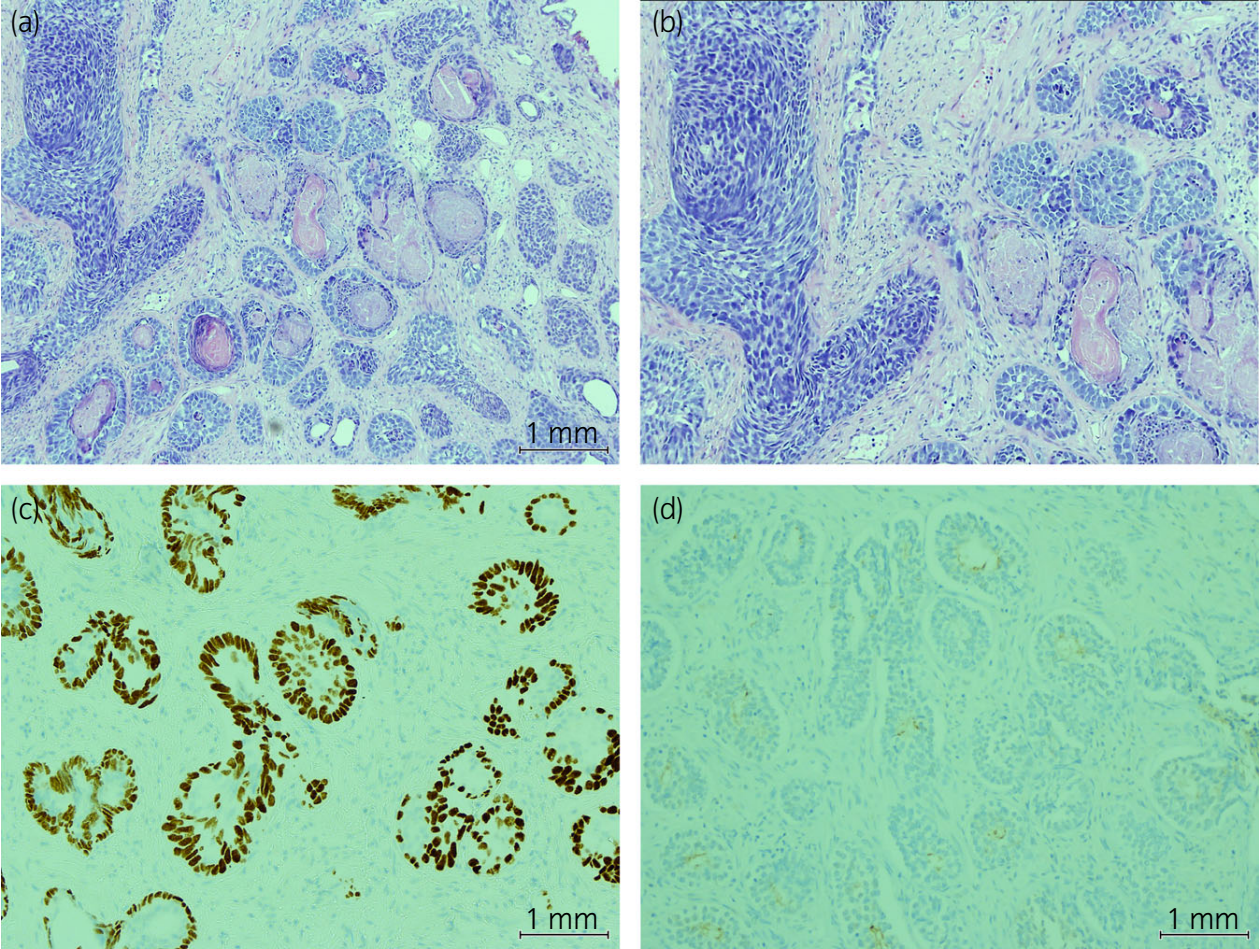
Histological examination of hemoxylin and eosin‐stained prostate tissue retrieved from TURP. (a) Multiple large and small nests of basal cell carcinoma and (b) neoplastic cells with prominent nucleoli in a palisading pattern, with areas necrosis and calcification. Tissue immunostaining demonstrated the neoplastic cells were (c) positive for p63 and (d) negative for PSA.

A 69‐year‐old man presented to the Urology Department with 2 years of lower urinary tract symptoms and macroscopic hematuria. His comorbidities included hypertension, hypercholesterolemia, and insulin‐dependent type 2 diabetes. Originally from Vietnam, he had a history of potential carcinogen exposure as a prisoner of war during the Vietnam War. Examination revealed normal external genitalia and a moderately enlarged, benign prostate on digital rectal exam, correlating with a gland volume of 61 cc on MRI. His PSA level and density was measured at 5.67 ng/mL and 0.09 ng/mL/m^3^. Subsequent bladder malignancy work up was normal, though an enlarged and vascular prostate was observed on flexible cystoscopy. After a failed trial of medical management, he underwent an elective TURP, in which 39.9 g of tissue was resected. Histology revealed a basaloid lesion with a spectrum of changes, including multiple foci of basal cell carcinoma. The cells were positive for P63, cytokeratin 5/6 and negative for alpha‐methylacyl‐CoA racemase immunohistochemistry. There was strong staining seen with Bcl2 and luminal staining seen with cytokeratin 7 (Fig. 1).

**Figure 2:**
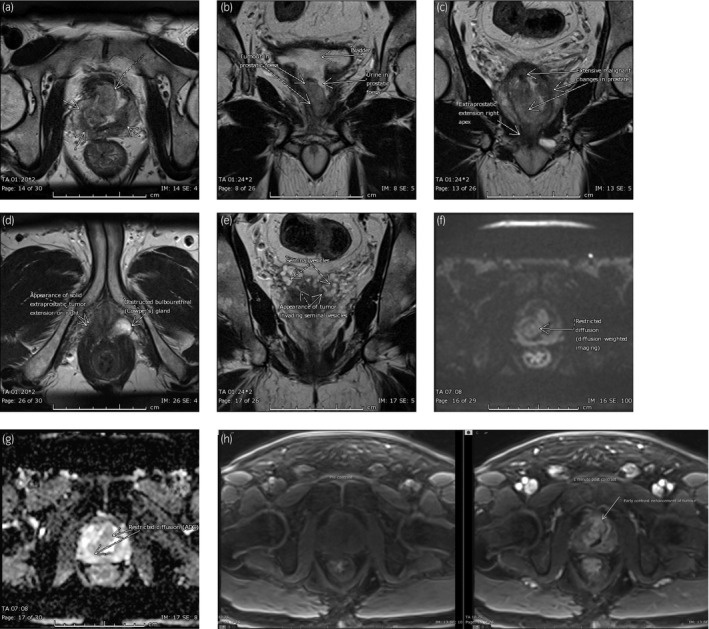
T2 weighted multiparametric MRI showing a bulky and extensive Prostate Imaging‐Reporting and Data System lesion involving the majority of the prostate, centered in the transitional zone and extending to the peripheral zone from base to apex (a, b). Arrows indicate the appearance of solid extra‐capsular extension on the right (c, d) and seminal vesicle involvement (e), not correlated on positron emission tomography/computed tomography. Standard MRI prostate protocol was performed at 3T, including axial DWI (b values 50, 800 and 1200) (f), ADC mapping (g) and dynamic post contrast‐enhanced imaging (h). The tumor demonstrated low to intermediate T2 signal with moderate diffusion restriction and early contrast enhancement with washout.

**Figure 3:**
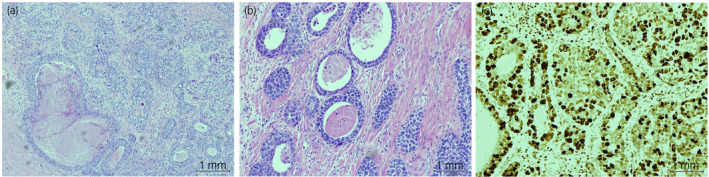
Examination of hematoxylin and eosin‐stained surgical specimen of the prostate. (a) Basal cell carcinoma with malignant cells arranged into irregular trabeculae, cords, and large nests with evidence of tumor necrosis. (b) The neoplastic cells have enlarged, pleomorphic, round, and oval nuclei, and are surrounded by scant basophilic cytoplasm. (c) Ki‐97 immunostain index was 50–90% in these areas.

**Figure 4:**
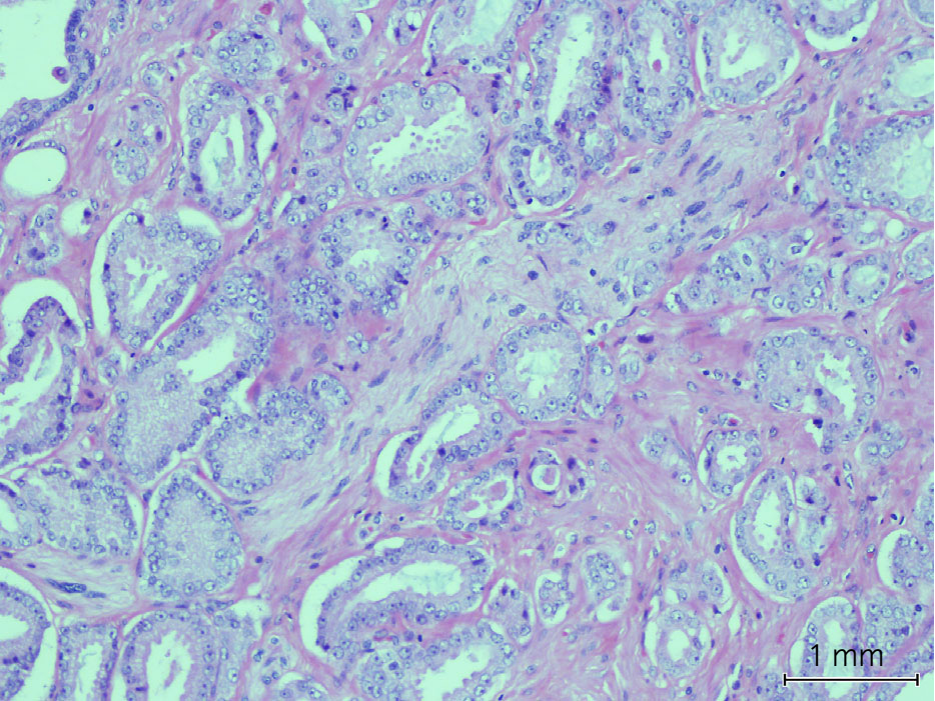
Foci of well differentiated prostatic acinar adenocarcinoma. Single layer of neoplastic cells arranged into separate glands of variable size and shape, with one focus of perineural invasion imaged above.

Subsequent staging investigations included multiparametric MRI of the prostate, which showed a bulky Prostate Imaging‐Reporting and Data System tumor, with suspicious extra‐capsular extension and seminal vesical involvement (Fig. 2). However, positron emission tomography‐computed tomography did not demonstrate fluorodeoxyglucose‐avidity within these areas, the surrounding pelvic lymph nodes or more distally. The patient was offered robot‐assisted radical prostatectomy. The final histopathology of the resected specimen showed basal cell carcinoma in both lobes, involving approximately 4% of the gland, without extra capsular extension, lymphovascular invasion, or seminal vesicle involvement (T2c) (Fig. 3). Concomitant acinar adenocarcinoma (Gleason 3 + 3) was also identified, involving less than 1% of the gland, with one focus of perineural invasion (Fig. 4). The PSA reduced to <0.03 ng/mL at 3 weeks post‐robot‐assisted radical prostatectomy. A follow‐up surveillance plan of 6‐monthly computed tomography abdomen/pelvis and PSA level was unanimously decided upon in consultation with urologists, uropathologists, and oncologists, given the unpredictable risk of recurrence in this disease.

## Discussion

The histology of healthy prostate glandular tissue comprises acini and ducts lined by secretory epithelium. Beneath the secretory lining is a layer of basal cells as well as interspersed endocrine‐paracrine cells.^1^ Basal cell carcinoma of the prostate was first reported in 1974, and can be classified as adenoid cystic or basaloid cell origin.^2^ Histologically, infiltrating basaloid cells form dilated acinar cribriform spaces with admixed glandular or trabecular material.^2^, ^3^ Tumors predominate in the transitional zone of the prostate, leading to early symptomatic bladder outlet obstruction and an enlarged and indurated gland on digital rectal examination.^1^ Both subtypes of PBCC have been found with co‐existent acinar adenocarcinoma.^4^ Serum PSA is usually low or normal, however, it can be elevated in patients who also have acinar adenocarcinoma.^5^ The diagnosis is often made incidentally during transurethral resection or enucleation performed for bladder outlet obstruction, though it is also discovered on needle biopsy and radical prostatectomy performed for biopsy‐positive conventional adenocarcinoma.^5^

In our case, basaloid cell carcinoma was identified on histopathological examination of a TURP specimen. Similar to many cases previously described in the literature, the basaloid lesion demonstrated a spectrum of changes, from relatively indolent cellular atypia to solid nests of carcinoma with necrosis and peripheral palisading. A pre‐operative PSA of 5.67 ng/mL was consistent with concomitant Gleason 3 + 3 acinar adenocarcinoma that was discovered on histological analysis of the surgical specimen (Fig. 4).

A recent case report published by Shibuya *et al*.^6^ yielded 98 cases of PBCC upon searching the PubMed electronic database. A wider search of the literature by our group revealed a total of 139 cases, of which 102 cases were published clinical reviews.^1^, ^2^, ^3^, ^4^, ^5^, ^6^, ^7^, ^8^, ^9^, ^10^, ^11^, ^12^, ^13^, ^14^, ^15^, ^16^, ^17^, ^18^, ^19^, ^20^ When summarizing these cases, the mean age at PBCC diagnosis was 67.9 ± 11.6, and the mean period of follow‐up was 37.7 ± 37.3 months. The most common primary complaint for these men was urinary obstruction. Of 102 cases, 63 (61.8%) proceeded to transurethral resection with incidental diagnoses of PBCC. Seventeen (16.7%) men were diagnosed after needle biopsy.

Radical prostatectomy was performed in 26 out of the 102 patients; whilst other treatment methods included conservative management, chemoradiotherapy, hormone therapies and pelvic exenteration. Of the patients diagnosed with PBCC, 25 (24.5%) developed locoregional recurrence or distant metastases, out of which nine (40.9%) died. However, a large proportion of patients had comparatively indolent disease, with 45 of 102 (44%) having no evidence of recurrence and nine (8.8%) having died of other causes. Concomitant adenocarcinoma was discovered on the formal histopathology of 13 patients, which did not appear to correlate with progression of disease.

At present, our knowledge of the natural history, grading and prognosis of PBCC is limited by its relatively low incidence. The literature demonstrates a range of presentations, with a small proportion of disease presenting as aggressive with a high risk of locoregional recurrence and mortality. Some studies have suggested high grade disease is more likely with basaloid cell‐predominant tumors.

The current case was discussed in a multidisciplinary setting, wherein pathologists and oncologists strongly recommended six‐monthly computed tomography of the abdomen and pelvis, and repeat serum PSA. This advice was based upon the most recent literature, which suggested an overall disease progression rate of 37% (metastasis or recurrence).^6^ More stringent risk stratification the time of PBCC diagnosis is crucial to determining appropriate prognostication and surveillance.

PBCC is a diagnosis which presents a difficult uro‐oncological challenge. The present case report and latest literature review suggest that more cases of PBCC are indolent than aggressive, though there currently exists no method of differentiation between high‐grade and low‐grade disease. As such, we recommend radical surgery for cases of PBCC with high histological grade or predominant basaloid component. Furthermore, our group recommends prudent surveillance for loco‐regional recurrence and distant metastases with half‐yearly computed tomography of the abdomen/pelvis and repeat PSA.

## Conflict of interest

The authors declare no conflict of interest.
